# Anticonvulsant Effects of Topiramate and Lacosamide on Pilocarpine-Induced Status Epilepticus in Rats: A Role of Reactive Oxygen Species and Inflammation

**DOI:** 10.3390/ijms22052264

**Published:** 2021-02-25

**Authors:** Michaela Shishmanova-Doseva, Lyudmil Peychev, Lyubka Yoanidu, Yordanka Uzunova, Milena Atanasova, Katerina Georgieva, Jana Tchekalarova

**Affiliations:** 1 Department of Pharmacology and Drug Toxicology, Medical University-Plovdiv, 4002 Plovdiv, Bulgaria; peych@propolisbg.com; 2 Department of Bioorganic Chemistry, Medical University-Plovdiv, 4002 Plovdiv, Bulgaria; lubka.yoanidu@gmail.com (L.Y.); d_anny@abv.bg (Y.U.); 3 Department of Biology, Medical University of Pleven, 5800 Pleven, Bulgaria; milenaar2001@yahoo.com; 4 Department of Physiology, Medical University-Plovdiv, 4002 Plovdiv, Bulgaria; kng@plov.net; 5 Institute of Neurobiology, Bulgarian Academy of Sciences (BAS), 1113 Sofia, Bulgaria

**Keywords:** status epilepticus, anticonvulsants, oxidative stress, IL-1β, TNF-α, hippocampus

## Abstract

Background: Status epilepticus (SE) is a neurological disorder characterized by a prolonged epileptic activity followed by subsequent epileptogenic processes. The aim of the present study was to evaluate the early effects of topiramate (TPM) and lacosamide (LCM) treatment on oxidative stress and inflammatory damage in a model of pilocarpine-induced SE. Methods: Male Wistar rats were randomly divided into six groups and the two antiepileptic drugs (AEDs), TPM (40 and 80 mg/kg, i.p.) and LCM (10 and 30 mg/kg, i.p.), were injected three times repeatedly after pilocarpine administration. Rats were sacrificed 24 h post-SE and several parameters of oxidative stress and inflammatory response have been explored in the hippocampus. Results: The two drugs TPM and LCM, in both doses used, succeeded in attenuating the number of motor seizures compared to the SE-veh group 30 min after administration. Pilocarpine-induced SE decreased the superoxide dismutase (SOD) activity and reduced glutathione (GSH) levels while increasing the catalase (CAT) activity, malondialdehyde (MDA), and IL-1β levels compared to the control group. Groups with SE did not affect the TNF-α levels. The treatment with a higher dose of 30 mg/kg LCM restored to control level the SOD activity in the SE group. The two AEDs, in both doses applied, also normalized the CAT activity and MDA levels to control values. In conclusion, we suggest that the antioxidant effect of TPM and LCM might contribute to their anticonvulsant effect against pilocarpine-induced SE, whereas their weak anti-inflammatory effect in the hippocampus is a consequence of reduced SE severity.

## 1. Introduction

Status epilepticus (SE) is a clinical condition characterized by prolonged or short-term but repeated seizures activity [1]. It results in epileptogenesis with devastating plastic changes in vulnerable brain structures, including decreased seizure threshold and neuronal injury [2,3]. SE may develop in already diagnosed epilepsy patients, due to the pathological hypersynchronized activity and the excitability of the neurons [4]. Brain trauma, infections, ischemia/hypoxia, cerebrovascular diseases, and febrile conditions have been pointed out amongst the leading causes for the de novo development of this condition [5]. A variety of mechanisms including enhanced neuroinflammatory response and overproduction of reactive oxygen and nitrogen species are involved in the pathophysiology of SE and subsequent epileptogenic processes [6].

It has been suggested that seizure generation could be provoked by a disturbed prooxidant/antioxidants ratio, leading to oxidative stress [7]. Production of free oxygen species at a physiological level is an essential part of normal cell metabolism. Reactive oxygen species (ROS), such as the superoxide radical, hydroxyl radical, singlet oxygen, as well as subsequently produced reactive nitrogen species (peroxynitrite anion), have been scavenged by several antioxidant systems. These could be either enzymatic systems—catalase (CAT), superoxide dismutase (SOD), glutathione peroxidase (GPx), glutathione reductase (GR)—or nonenzymatic antioxidants—reduced glutathione (GSH) and vitamins C and E [7]. Brain tissue is vulnerable to oxidative injury for its high oxygen saturation, high content of polyunsaturated fatty acids and iron, as well as low CAT activity [8]. All the factors mentioned above contribute so that oxidative stress easily damages neurons.

Increased release of cytokines, such as interleukin-1beta (IL-1β), tumor necrosis factor-α (TNF-α), and interleukin-6 (IL-6), in the brain tissue during SE can affect the excitability of the central nervous system and lead to neuronal dysfunction and loss of neuronal cells [9]. These inflammatory mediators are mainly produced by the activated glial cells and play a key role in the pathophysiological relationships between reactive microglia and astrocytes, and neuronal and endothelial cells [4]. Inflammation-signaling molecules, IL-1β and TNF-α, can increase the calcium and sodium currents in the hippocampal neuronal cultures and modulate their distribution in neuronal membranes [10]. They can also enhance the expression of AMPA receptors and NMDA-receptor-mediated responses [5,11]. Besides, pro-inflammatory cytokines inhibit the astrocytic reuptake of glutamate, and this process leads to abnormal extracellular glutamate levels [6]. On the other hand, IL-1β and TNF-α can attenuate classical inhibitory GABAergic neurotransmission [5,11]. All these changes could increase the neuronal network excitability and diminish cell survival and seizure threshold. These data correspond to clinical and experimental studies in which there have been increased levels of IL-1β and TNF-α demonstrated in patients with temporal lobe epilepsy and humans exposed to SE [4,12], as well as in animals with SE [13].

Different experimental models with chemoconvulsants, such as kainic acid (KA), pilocarpine (Pilo), or pentylenetetrazol (PTZ), have been used to describe the specific mechanisms, the effect of applied drug therapy, as well as the possibility of preventing neuronal damage after acute seizure activity. Topiramate (TPM) is a second-generation antiepileptic drug (AED) effective against a broad spectrum of seizure types. It exerts anticonvulsant action by reducing voltage-dependent sodium and l-type calcium channels, increasing Cl- influx via GABAA receptor, and inhibiting the release of glutamate and aspartate through AMPA receptors [14]. This AED exhibits disease-modifying activity in preclinical studies [15]. Lacosamide (LCM) is a third-generation AED approved for focal epilepsy treatment. The suppression of voltage-gated sodium channels has been proposed to be a main mechanism of the drug anticonvulsant activity [16]. Although the pharmacological activity of these drugs has been well studied, little is known about the effects of TPM and LCM on oxidative stress and neuroinflammation, outlined as major contributors in the subsequent processes of epileptogenesis. Therefore, in the present study, we aimed to explore the effects of the two AEDs, TPM and LCM, repeatedly injected during Pilo in rats concomitant to SE oxidative stress and neuroinflammation in the hippocampus.

## 2. Results

### 2.1. Number of Motor Seizures during SE

**Figure 1:**
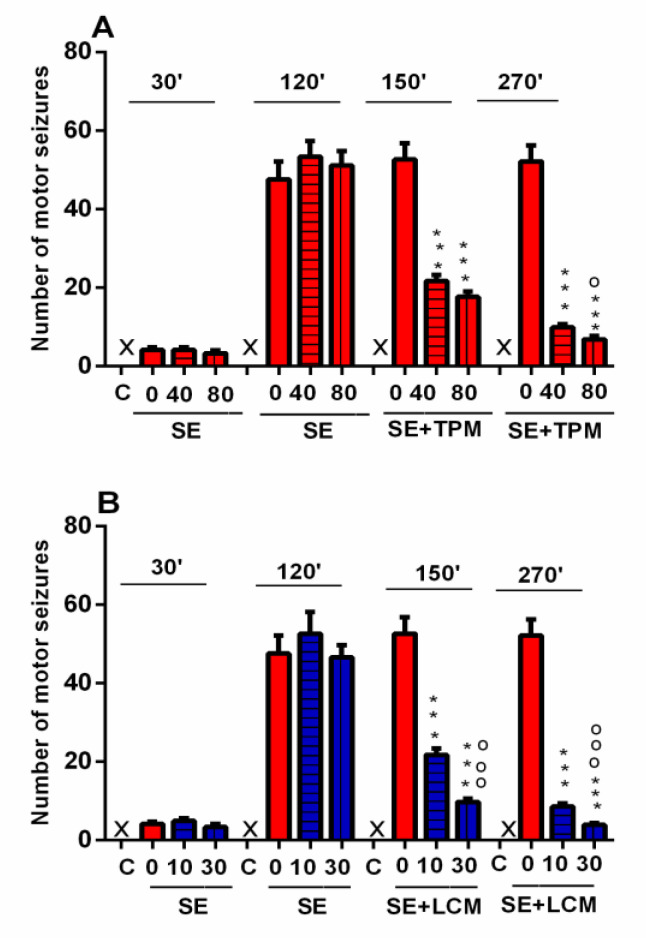
(**A**,**B**) Number of motor seizures at 30, 120, 150, and 270 min after induction of the status epilepticus (SE). (**A**) Effect of topiramate (TPM), 40 and 80 mg/kg, on the number of motor seizures 150 and 270 min after the onset of SE. Mixed model of one-way ANOVA (MANOVA) showed that the Mauchly’s test of sphericity assumption was violated (χ^2^ (5) = 112.872, *p* < 0.001) for TPM treatment. Therefore, degrees of freedom were corrected using Greenhouse–Geisser estimates of sphericity (ε = 0.537). The analysis revealed a significant main effect of Time [F(1.612, 83.798) = 98.618, *p* < 0.001] and Time × Treatment interaction for TPM [F(1.612, 83. 798) = 45.970, *p* < 0.001]. *** *p* < 0.001 vs. SE-veh group; ^o^ *p* < 0.05 vs. SE-TPM-40 group. (**B**) Effect of lacosamide (LCM), 10 and 30 mg/kg, on the number of motor seizures 150 and 270 min after the onset of SE. Mixed model of one-way ANOVA showed that the Mauchly’s test of sphericity assumption was violated (χ^2^ (5) = 103.893, *p* < 0.001) for LCM treatment. Therefore, degrees of freedom were corrected using Greenhouse–Geisser estimates of sphericity (ε = 0.546). The analysis revealed a significant main effect of the time [F(1.637, 83.464) = 84.020, *p* < 0.001] and Time × Treatment interaction for data with LCM [F(1.637, 83.464) = 43.704, *p* < 0.001]. *** *p* < 0.001 vs. SE-veh group; ^ooo^ *p* < 0.001 vs. SE-LCM-10 group.

**Figure 2:**
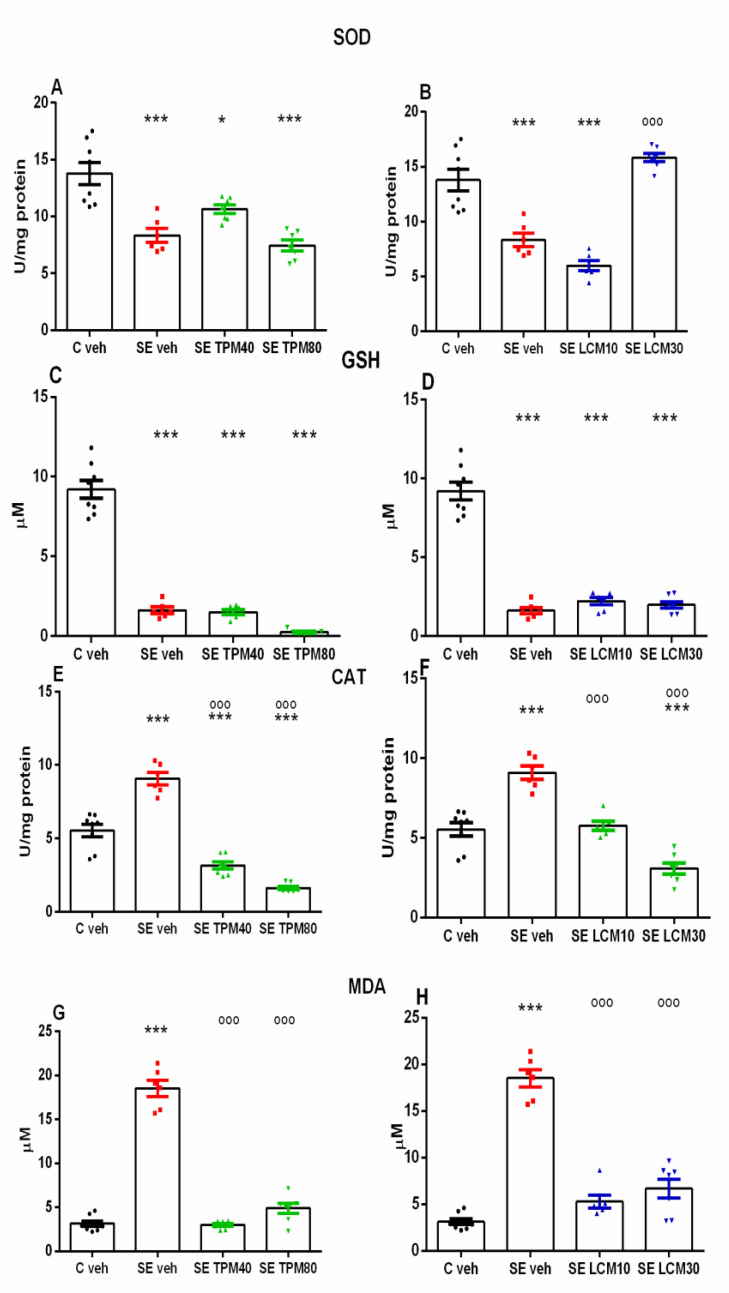
(**A**) Effect of TPM, 40 and 80 mg/kg, on superoxide dismutase (SOD) activity (U/mg protein) in rat hippocampus 24 h after SE (Kruskal–Wallis one-way ANOVA on ranks: H = 20.922, * *p* < 0.05; *** *p* < 0.001 vs. C-veh group); (**B**) effect of LCM, 10 and 30 mg/kg, on SOD activity 24 h after SE (Kruskal–Wallis analysis: H = 20.991, *** *p* < 0.001 vs. C-veh group; ^ooo^ *p* < 0.001 vs. SE-veh group, respectively); (**C**) effect of TPM, 40 and 80 mg/kg, on reduced glutathione (GSH) levels (µM) in rat hippocampus 24 h after the SE (Kruskal–Wallis analysis: H = 23.323, *** *p* < 0.001 vs. C-veh group); (**D**) effect of LCM, 10 and 30 mg/kg, on GSH levels 24 h after SE (Kruskal–Wallis analysis: H = 17.944, *** *p* < 0.001 vs. C-veh group); (**E**) effect of TPM, 40 and 80 mg/kg, on catalase (CAT) activity (U/mg protein) in rat hippocampus 24 h after SE (one-way ANOVA: F3,27 = 86.657; *** *p* < 0.001 vs. C-veh group; ^ooo^ *p* < 0. 001 vs. SE-veh group); (**F**) effect of LCM, 10 and 30 mg/kg, on CAT activity 24 h after SE (one-way ANOVA: F3,27 = 38.921; *** *p* < 0.001 vs. C-veh group; ^ooo^ *p* < 0.001 vs. SE-veh group); (**G**) effect of TPM, 40 and 80 mg/kg, on malondialdehyde (MDA) levels (µM) in rat hippocampus 24 h after SE (one-way ANOVA: F3,27 = 183.305, *** *p* < 0.001 vs. C-veh group; ^ooo^ *p* < 0.001 vs. SE-veh group); (**H**) effect of LCM, 10 and 30 mg/kg, on MDA levels 24 h after SE (one-way ANOVA: F3,27 = 183.305, *** *p* < 0.001 vs. C-veh group; ^ooo^ *p* < 0.001 vs. SE-veh group).

No significant difference in the number of motor seizures has been detected among the three groups injected with pilocarpine at the 30th and 120th min after SE induction (Figure 1A,B). The two AEDs alleviated the seizure frequency compared to the SE-veh group at the 150th min, i.e., thirty minutes after the first injection, as well as at the 270th min after SE, at doses of 40 and 80 mg/kg for TPM (*p* < 0.001), and at doses of 10 and 30 mg/kg for LCM (*p* < 0.001), respectively. At the 150th min after SE, the number of seizures was significantly lower for the group with a higher dose of LCM SE-LCM-30 when compared to the SE-LCM-10 group (*p* < 0.001) (Figure 2B). Further, at the 270th min after SE, the two AEDs with the higher dose, SE-TPM-80 and SE-LCM-30, respectively, had fewer seizures than the two groups with lower doses, SE-TPM-40 and SE-TPM-10 (*p* < 0.001) (Figure 1A,B).

### 2.2. Biomarkers of Oxidative Stress in the Hippocampus

Status epilepticus caused a significant decrease in SOD activity in the SE-veh group compared to the C-veh group (*p* < 0.001) (Figure 2A,B). The repeated treatment with TPM during SE was unable to restore the SE-induced diminished SOD activity to control level at the two doses tested, 40 and 80 mg/kg, respectively (*p* < 0.001 compared to C-veh group), while the enzyme activity was found to be significantly increased by LCM at a dose of 30 mg/kg compared to the SE-veh group (*p* < 0.001) (Figure 2B).

**Figure 3:**
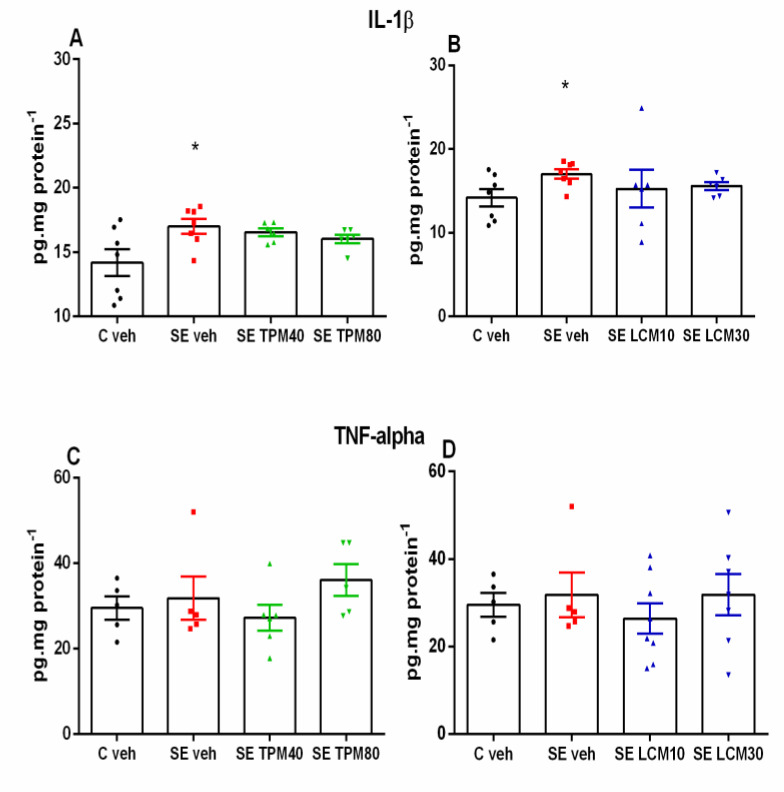
(**A**) Effect of TPM, 40 and 80 mg/kg, on IL-1β levels (pg.mg protein-1) in rat hippocampus 24 h after SE (one-way ANOVA: F3,22 = 3.607, * *p* < 0.05 vs. C-veh group); (**B**) effect of LCM, 10 and 30 mg/kg, on IL-1β levels 24 h after SE (one-way ANOVA: F3,22 = 3.75, * *p* < 0.05 vs. C-veh group); (**C**) effect of TPM, 40 and 80 mg/kg, on TNF-α levels (pg.mg protein-1) in rat hippocampus 24 h after SE; (**D**) effect of LCM, 10 and 30 mg/kg, on TNF-α levels 24 h after SE.

The GSH level was found to be significantly decreased in the SE-veh group in comparison with the C-veh group (*p* < 0.001) (Figure 3C,D). However, neither TPM, at doses of 40 and 80 mg/kg, nor LCM, at doses of 10 and 30 mg/kg, respectively, was able to reverse the GSH level to the control value (*p* < 0.001 compared to the C-veh group) (Figure 2C,D).

The post-hoc test demonstrated that the SE-veh group had a significantly higher CAT activity compared to C-veh (*p* < 0.001) (Figure 2E,F). The CAT activity was demonstrated to be significantly diminished by treatment with the two AEDs, TPM, at doses of 40 and 80 mg/kg, and LCM, at doses of 10 and 30 mg/kg, respectively, compared to the C-veh and SE-veh group, respectively (*p* < 0.001) (Figure 2E,F).

The post-hoc test showed that the SE-veh group significantly elevated the MDA levels compared to the C-veh group (*p* < 0.001), while the repeated treatment by both TPM, at doses of 40 and 80 mg/kg, and LCM, at doses of 10 and 30 mg/kg, after SE significantly alleviated the lipid peroxidation compared to the SE-veh group (*p* < 0.001) (Figure 2G,H).

### 2.3. IL-1β and TNF-α Levels in the Hippocampus

The pilocarpine-induced SE produced a significant elevation in IL-1β level in the SE-veh group compared to the C-veh group (*p* < 0.001) (Figure 3A,B). The two AEDs partially affected the SE-related surge in IL-1β at the two doses used (Figure 3A,B).

For the TNF-α levels, no significant difference was observed among the groups C-veh, SE-veh, and that treated with either TPM (40 and 80 mg/kg) or LCM (10 and 30 mg/kg) (*p* > 0.05) (Figure 3C,D).

## 3. Discussion

In the present study, we found that the two AEDs, mainly used as the 2nd and 3rd line drugs in clinical practice, and given repeatedly, after the onset of SE, at doses of 40 and 80 mg/kg, for TPM, and at doses of 10 and 30 mg/kg, for LCM, respectively, suppressed behavioral motor seizures and accompanying oxidative stress, and partially mitigated SE-induced neuroinflammation in the hippocampus.

In some patients, prolonged SE may be refractory to drug treatment and later develop into chronic epilepsy. On the other hand, SE may be an intrinsic manifestation of the disease, sometimes with recurrent episodes [17]. In the present study, the two AEDs, TPM and LCM, characterized by different mechanisms of action, showed a similar potency of mitigating the Pilo-induced SE, in a dose-dependent manner, immediately after the first injection at the 120th min after a SE onset. TPM is effective in the suppression of SE in both patients and animal models [18,19].

Although LCM is not approved for SE treatment, accumulated clinical and preclinical data support the suggestion that LCM adjunctive therapy with standard AEDs might be successfully applied in refractory SE [20] and even be able to replace them [21,22,23]. Moreover, clinical evidence has shown that LCM has been effective in nonconvulsive and generalized convulsive SE treatment, as well as in focal motor SE management [23]. This calls for a detailed exploration of LCM in animal models of SE. The present results agree with those of our previous experiment in which the repeated LCM administration suppressed the KA-induced SE and epileptogenesis in rats [24]. These data coincide with Nirwan et al. [25] who also proved that LCM can mitigate the Pilo-induced SE in mice at doses of 20 and 40 mg/kg, respectively. The anticonvulsant and neuroprotective potency of LCM is due to its ability to suppress the interictal spike rates, the high-frequency oscillations in the hippocampus, as well as the mossy fiber sprouting and the loss of hippocampal neurons [26,27].

ROS play an important role in the cell signaling cascade. The impaired ROS neutralization was demonstrated through increased lipid peroxidation, and decreased GSH level and SOD activity, while the adaptive elevation of CAT activity was detected in the hippocampus 24 h after SE onset in rats.

SOD is an enzyme that catalyzes the conversion of the superoxide radical O^2−^ to H_2_O_2_, which in turn, is neutralized by CAT. SOD overexpression can provide neuroprotection against the deleterious effects of a wide range of brain disorders including epilepsy [28]. Here, we report that LCM at its higher dose of 30 mg/kg managed to increase the enzyme activity, which in turn, could protect proteins from oxidation and reduce undesired structural and functional changes in some key enzymes. Our present findings in the Pilo-induced SE rat model and those of Nirwan et al. [25] in the same model in mice suggest that the antioxidant effect of LCM is an important mechanism underlying the ability of this AED to mitigate SE and its devastating consequences. Nirwan et al. [25] reported that LCM, administered at the same doses used as in our experimental protocol, exerted a biphasic activity with a potent anticonvulsant, antioxidant, and neuroprotective effect against Pilo-induced SE in mice. However, these authors revealed that LCM was inactive at a high dose of 80 mg/kg against both recurrent seizures and concomitant oxidative stress as well. Like LCM, the AED oxcarbazepine, administered in a higher dose, exerted pro-oxidant and neurotoxic activity [29]. TPM was unable to correct the SE-induced decrease in SOD activity in the rat hippocampus. By contrast, Mazhar et al. [30] reported that TPM is one of the most potent AEDs able to enhance the SOD activities in the PTZ kindled mice, suggesting that its scavenging activity is responsible for the anticonvulsant effect. This discrepancy with our findings might be explained mostly with model- and strain-related differences, which lead to a diverse TPM impact on enzyme activity.

GSH is a nonenzymatic antioxidant tripeptide, which plays a crucial role in the central nervous system defense from ROS overproduction, both in intra- and extracellular medium. During excessive ROS production, the reduced GSH is consumed and the oxidized form has been found to prevail [31]. However, we demonstrated that the two AEDs, TPM and LCM, failed to prevent SE-induced impaired GSSG/GSH balance and decreased the level of the antioxidant biomarker GSH. Mazhar et al. [30] report that TPM restores the levels of GSH in PTZ-kindled mice and suggests a direct connection between the AED-associated anticonvulsant effect and the levels of ROS. Nirwan et al. [25] also show that LCM dose-dependently increases the GSH level in the hippocampus of mice subjected to Pilo-induced SE. The divergence in these results may be explained by the different strain (rats vs. mice), the different doses of LCM (30 vs. 40 mg/kg), and the different times of decapitation after SE (24 vs. 48 h).

CAT is an enzymatic antioxidant system involved in the scavenging of H_2_O_2_. The activity of this enzyme is low in the brain and that is one of the reasons why this organ becomes susceptible to oxidative damage [32]. Several studies have demonstrated different changes in CAT activity during SE. These seem to be strictly dependent on the mechanisms of the different convulsants that evoke seizure induction [33,34]. In agreement with Freitas et al. [35,36] and Santos et al. [37], we report that Pilo-induced SE is accompanied by an adaptive elevation of CAT activity in the hippocampus, which may be due to the excessive production of ROS during the ongoing seizures.

Literature data have shown that AEDs such as TPM and levetiracetam can induce lipid peroxidation and impair the antioxidant defense system in naive rats, while the same drugs produce a disease-modifying effect with antioxidant potency both in patients and experimental models of acquired epilepsy [38,39,40]. Moreover, new AEDs such as lamotrigine and oxcarbazepine may have pronounced beneficial effects in epileptic and nonepileptic conditions while the first-generation medications such as phenytoin, phenobarbital, and carbamazepine may trigger additional oxygen-dependent tissue injury in epileptic patients [41,42]. The pro-oxidant effects of the 1st line AEDs could worsen the progression of the disease, leading to cognitive dysfunction and other comorbidities such as depression- and anxiety-like symptoms accompanied by a loss of AEDs efficacy [43]. Excessive release of ROS can interact with polyunsaturated fatty acids leading to lipid peroxidation, which occurs with the production of MDA. The treatment with either TPM or LCM in the two doses used prevented the brain from lipid peroxidation, suggesting a beneficial role in oxidative damage-related processes. The favorable effect of TPM on lipid peroxidation induced in PTZ-kindled mice may be explained with the attenuation of glutamate-mediated neurotoxicity, which decreases the ROS production [30]. In compliance with our results, Sairazi [44] demonstrated a decreased lipid peroxidation and increased total antioxidant status 24 h after a KA-induced model of SE in rats pre-treated with TPM. LCM has also been shown to reverse the brain lipid peroxidation and to increase the SOD activity in a strychnine-induced seizure model [33]. Besides, LCM attenuates both the MDA levels in serum and the glial activation in a model of traumatic spinal cord injury [45].

In the last decades, accumulated research data have been elucidating the pivotal role of immune and inflammatory processes in the pathogenesis of seizures in various types of epilepsy, as well as during SE. The rapid neuroinflammatory response during SE is involved in the onset and the spread of seizure activity in different brain areas [46]. The increased biosynthesis and release of IL-1β and TNF-α activate the cytokine receptors in neurons and rapidly alter their excitability, which has been believed to play a crucial role in their epileptogenic effect, observed in both clinical and experimental studies [47,48]. In our study, we found that an inflammatory process was activated in the hippocampus via the increase in the pro-inflammatory cytokine IL-1β. However, the TNF-α level reached the control level 24 h after the onset of SE. Our findings comply with literature data that hippocampal levels of IL-1β have remained high 24 h after SE. However, the peak level for TNF-α was reported to be around 5–7 h after SE and then to return to basal level [12,47]. The anticonvulsant effect of the IL-1β signaling pathway blockade has been shown in rat electrical kindling [49]. Likewise, suppression of TNF-α level has been associated with the decreased epileptic activity. However, no significant change of this cytokine in plasma or cerebrospinal fluid has been reported in patients 24 h after acute tonic-clonic seizures or after febrile seizures [50,51]. The present data indicated that TPM and LCM produced no significant effect on cytokines. This result agrees with previous reports demonstrating the potency of TPM and LCM against lipopolysaccharide-induced inflammation both in vitro and in vivo [52,53]. However, literature data for a direct anti-inflammatory effect of the two AEDs in seizure models are lacking and further experiments are needed to elucidate the precise role of the inflammatory process in the activity of TPM and LCM.

## 4. Materials and Methods

### 4.1. Reagents

Phenazine methosulfate (PMS), nitroblue tetrazolium (NBT), trichloroacetic acid (TCA), thiobarbituric acid (TBA), 5,5′-dithio-bis-(2-nitrobenzoic acid) (DTNB), pilocarpine hydrochloride (Pilo), and scopolamine methyl nitrate were purchased from Sigma Aldrich (Hamburg, Germany). LCM (Vimpat, USB Pharma, Brussels, Belgium); TPM (Topamax, Janssen-Pharmaceutica NV, Beerse, Belgium); diazepam (Sopharma, Sofia, Bulgaria); IL-1 beta Rat ELISA Kit—Invitrogen (Thermo Fisher Scientific, Vienna, Austria). TNF alpha Rat ELISA Kit—Invitrogen, Thermo Fisher Scientific.

### 4.2. Animals

This study was performed in strict accordance with the guidelines of the European Community Council directives 86/609/EEC. 0.2010/63/EC. Experiments were approved by the Bulgarian Food Safety Agency № 206/1 October 2018 and by the Ethical Committee on Human and Animal Experimentation of Medical University—Plovdiv №1/28 February 2019. Ninety-six mature male Wistar rats (3 months of age) with body weights ranging from 180 to 200 g were used in the experiment. They were obtained from the Animal Center of Medical University—Plovdiv. The rats were housed in plastic cages (5–6 per cage) in a temperature- and humidity-controlled room with a 12/12 h light/dark cycle. Food and drinking water were allowed ad libitum.

The rats were randomly divided into 6 groups (*n* = 10 in each group): Group 1—controls (C-veh), treated with saline (1 mL/kg p.os); group 2—epilepsy group (SE-veh), treated with saline (1 mL/kg i.p.) and with induced SE with Pilo 320 mg/kg i.p.; group 3—epilepsy and TPM (SE-TPM-40), treated with TPM 40 mg/kg i.p. and induced SE with Pilo 320 mg/kg i.p.; group 4—epilepsy and TPM (SE-TPM-80), treated with TPM 80 mg/kg i.p. and with induced SE with Pilo 320 mg/kg i.p.; group 5—epilepsy and LCM (SE-LCM-10), treated with LCM 10 mg/kg i.p. and with induced SE with Pilo 320 mg/kg i.p.; and group 6—epilepsy and LCM (SE-LCM-30), treated with LCM 30 mg/kg i.p. and with induced SE with Pilo 320 mg/kg i.p.

### 4.3. Experimental Design and Induction of Status Epilepticus

**Figure 4:**
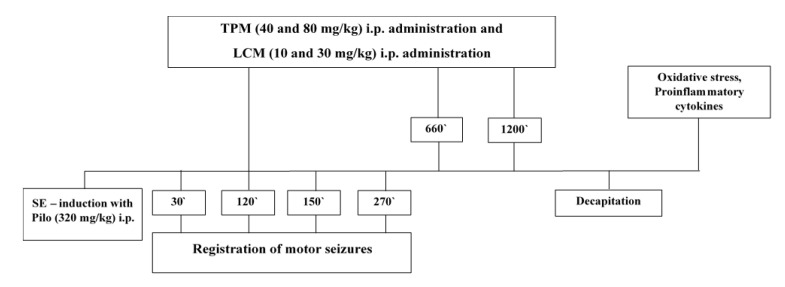
Schematic diagram of the experimental protocol.

The experimental design is demonstrated in Figure 4. Rats from 5 groups with SE were injected with Pilo hydrochloride 30 min after the injection of scopolamine methyl nitrate 1 mg/kg i.p., applied to reduce the peripheral cholinergic effects. After the Pilo administration, all the animals were placed in separate cages. Seizures appeared 15–25 min after Pilo injection and their behavior was classified using the Racine scale as Stage 0—no response, no tremor or seizures; Stage I—facial clonus (rhythmic chewing, blinking, moving, etc.); Stage II—Stage I plus rhythmic nod; Stage III—Stage II plus forelimb myoclonus without upright hind limbs; Stage IV—Stage III plus upright hind limbs; Stage V—generalized tonic, a burst of seizures and loss of position control. SE is defined as two or more seizures within a 5 min period without returning to normal between them or as a single seizure lasting for more than 5 min [17]. The criteria for reaching SE were a state with sustained convulsive seizures of stage III, IV, and V. After two hours of ongoing seizures, the rats received either TPM (40 and 80 mg/kg) or LCM (10 and 30 mg/kg) i.p. These doses were repeated twice more during the next 24 h at 9 h intervals. The number of motor seizures of stage III, IV, and V were calculated for each group at the 30th, 120th, 150th, and 270th min after the onset of SE. All animals were decapitated 24 h post SE, after thiopental administration (30 mg/kg, i.p.). After, the hippocampi were isolated, frozen in liquid nitrogen, and later stored at −70 °C until further processing [54].

### 4.4. Biomarkers of Oxidative Stress in the Hippocampus

#### 4.4.1. Preparation of Tissue Homogenate

The hippocampus was homogenized in an ice bath with phosphate buffer (pH = 7) and a tissue:buffer ratio of 1:25. The homogenate was then centrifuged at 10,000× *g* for 5 min at 4 °C. The obtained supernatant was transferred into another test tube and used for the analysis of CAT and SOD activities, as well as for determining the concentrations of MDA and GSH.

#### 4.4.2. SOD Activity Assay

SOD activity was measured based on the method of Kakkar et al. [55]. The reaction mixture was composed of 600 µL 0.052 mM phosphate buffer (pH = 8.16), 50 µL PMS (186 µM), 150 µL NBT (300 µM), and 150 µL supernatant. The reaction was started by the addition of 100 µL NADH (780 µM) and was stopped after 1 min with 500 µL glacial acetic acid. Absorbance was then measured against a blank sample, at 560 nm. SOD activity was calculated as U/mg protein.

#### 4.4.3. CAT Activity Assay

CAT activity was measured using 0.036% solution of hydrogen peroxide in 50 mM phosphate buffer (pH = 7). Then, 100 µL of the supernatant was added to 2.9 mL of the hydrogen peroxide solution and the changes in absorbance were monitored for 3 min at 240 nm. CAT activity was calculated as U/mg protein.

#### 4.4.4. GSH Assay

GSH was measured according to Khan’s assay [56]. To 50 µL of the homogenate, 50 µL of 10% TCA was added. The mixture was centrifuged at 1500× *g* for 15 min and the supernatant was taken for analysis. To 200 µL, 262 mM Tris-HCl (pH = 8.9), 20 µL DTNB, and 50 µL of the supernatant were added. Absorbance was measured after a 15 min reaction time, against a blank sample at 412 nm.

#### 4.4.5. MDA Assay

MDA was measured according to Khan’s assay [56]. The reaction mixture composed of 290 µL 0.1 M phosphate buffer (pH = 7.4), 100 µL supernatant, 100 µL ascorbic acid (100 µM), and 10 µL iron trichloride (100 µM) was incubated for 1 h at 37 °C. At the end of the incubation time, 500 µL of each 10% TCA and 0.67% TBA were added. Test tubes were then placed in a boiling water bath for 20 min, taken out, and placed on ice and centrifuged at 2500× *g* for 10 min. The absorbance of the supernatant was measured against a blank sample at 535 nm.

#### 4.4.6. Protein Content

Protein content was determined by a method that employs the use of bicinchoninic acid as a reagent and bovine serum albumin as a standard. A calibration curve was built for the interval 25–1000 µg/mL and the protein concentration was calculated according to it. To 2 mL reagent, 100 µL supernatant was added and the mixture was incubated for 30 min at 37 °C. Absorbance was measured against a blank sample at 562 nm. All spectrophotometric measurements were conducted on a CARY 1 spectrophotometer (Varian, Victoria, Australia).

### 4.5. Measurement of IL-1β and TNF-α

After animal decapitation, the hippocampus and frontal cortex were isolated on ice and preserved at −20 °C until the performance of the biochemical tests. The tissue samples were homogenized in 10 mL/g tissue in cold buffer containing 10 mM Tris HCL (pH 7.6), 1 mM EGTA, 50 mM NaF, 1 mM EDTA, and 1 mM PMSF. The protein content of each sample was measured by the Bradford method [57]. The measurement of the TNF- α was performed with an Invitrogen ELISA kit after centrifugation at 12,000× *g*, 4 °C, for 10 min of the tissue homogenate, and the concentration was expressed as pg.mg protein-1. Following the described homogenization and centrifugation procedures, the levels of IL-1β were assayed with a kit (Invitrogen, Rat IL-1beta Coated ELISA kit, BMS630). Amounts of IL-1β were measured in pg/mg protein. Each sample was measured in duplicate and the average was calculated.

### 4.6. Statistical Analysis

The number of seizures was assessed by the mixed model of one-way ANOVA (MANOVA) where the within-subject factor was Time while Treatment was the between-subject factor. For the within-subject factor, Mauchly’s test of sphericity was applied to examine whether the assumption of sphericity was met (*p* > 0.05) or violated (*p* < 0.05). In the second case, to adjust the degrees of freedom of the numerator and denominator, the Greenhouse–Geisser adjustment factor (ε) was applied. One-way analysis of variance was applied for the evaluation of data with oxidative stress and inflammation. If a significant difference was detected, analysis of variance was followed by a post-hoc Bonferroni test. For not normally distributed data, ANOVA for nonparametric data (Kruskal–Wallis on ranks) followed by the Mann–Whitney U test was applied. All data were presented as mean ± mean standard error. SigmaStat^®^11.0 and SPSS 19 software packages were used for analysis and a *p* < 0.05 value was accepted as a significant difference. For comprehensive information, see supplemental source data.

## 5. Conclusions

The present study revealed that repeated treatment with either TPM or LCM managed to mitigate the SE in the two doses used in a rat model of pilocarpine-induced SE. While LCM corrected to control level the SOD activity in the SE group, both LCM and TPM restored the CAT activity and MDA levels to control values, suggesting that the antioxidant effects of TPM and LCM contribute to mitigation of epileptogenesis and diminished risk of devastating comorbidities. The weak anti-inflammatory potency of the two AEDs might be a consequence of reduced SE severity but not an important mechanism underlying seizure suppression. However, additional studies are needed in the future to determine whether the antioxidant effect is obligatory or concomitant to the anticonvulsant effects of TPM and LCM in SE.

## Abbreviations

AEDs	antiepileptic drugs
AMPA	α-amino-3-hydroxy-5-methyl-4-isoxazolepropionic acid
ANOVA	analysis of variance
CAT	catalase
DTNB	5,5′-dithio-bis-(2-nitrobenzoic acid
GABA	γ-aminobutyric acid
GPx	glutathione peroxidase
GR	glutathione reductase
GSH	reduced glutathione
IL-1β	interleukin-1β
IL-6	interleukin-6
KA	kainic acid
LCM	lacosamide
MDA	malondialdehyde
NBT	nitroblue tetrazolium
NMDA	*N*-methyl-d-aspartate
Pilo	pilocarpine
PMS	phenazine methosulfate
ROS	reactive oxygen species
SE	status epilepticus
SOD	superoxide dismutase
TBA	thiobarbituric acid
TCA	trichloroacetic acid
TNF-α	tumor necrosis factor-α
TPM	topiramate
